# Identification of key gene modules and genes in colorectal cancer by co-expression analysis weighted gene co-expression network analysis

**DOI:** 10.1042/BSR20202044

**Published:** 2020-09-01

**Authors:** Peng Wang, Huaixin Zheng, Jiayu Zhang, Yashu Wang, Pingping Liu, Xiaoyan Xuan, Qianru Li, Ying Du

**Affiliations:** 1 Department of Immunology, School of Basic Medical Sciences, Zhengzhou University, Zhengzhou 450001, China; 2 Infection, Inflammation and Immunity Center, The Academy of Medical Sciences of Zhengzhou University, Zhengzhou, China; 3 State Key Laboratory of Membrane Biology, University of Chinese Academy of Science, Beijing, China

**Keywords:** Co-expression, Colorectal cancer, Gene module, Hub gene, WGCNA

## Abstract

Colorectal cancer (CRC) has been one of the most common malignancies worldwide, which tends to get worse for the growth and aging of the population and westernized lifestyle. However, there is no effective treatment due to the complexity of its etiology. Hence, the pathogenic mechanisms remain to be clearly defined. In the present study, we adopted an advanced analytical method—Weighted Gene Co-expression Network Analysis (WGCNA) to identify the key gene modules and hub genes associated with CRC. In total, five gene co-expression modules were highly associated with CRC, of which, one gene module correlated with CRC significantly positive (R = 0.88). Functional enrichment analysis of genes in primary gene module found metabolic pathways, which might be a potentially important pathway involved in CRC. Further, we identified and verified some hub genes positively correlated with CRC by using Cytoscape software and UALCAN databases, including *PAICS, ATR, AASDHPPT, DDX18, NUP107* and *TOMM6*. The present study discovered key gene modules and hub genes associated with CRC, which provide references to understand the pathogenesis of CRC and may be novel candidate target genes of CRC.

## Introduction

Besides lung cancer and breast cancer, colorectal cancer (CRC) is the most commonly diagnosed malignant cancer worldwide, which accounts for over 9% of all cancer incidences [1]. Meanwhile, it is also the fourth leading cause of cancer deaths in men and the third in women in the world, which accounted for nearly 8% of all cancer deaths in 2012 [1–3]. CRC has been a major public health problem and a huge burden worldwide, which is estimated to increase more than 2.2 million new cases and 1.1 million deaths by 2030 [4]. It has been recognized that CRC risks are closely associated with environmental factors and gene mutations. However, the pathogenesis of CRC has not been well revealed, as it is a heterogeneous disease associated with multiple genes and pathways. Deep and systematic understanding of genetic etiology and pathology of CRC, which may find new candidate target genes and provide new potential treatment strategies and methods.

Correlation networks are increasingly used in biology and medical sciences. Weighted Gene Co-expression Network Analysis (WGCNA) is an advanced systems biology-based methodology for exploring molecular mechanisms [5]. By constructing gene–gene co-expression networks to find clusters of highly correlated genes (module) and linking the information to measured traits, WGCNA has been widely and successfully used to identify candidate biomarkers and therapeutic targets in various complicate diseases [5]. For example, by using WGCNA method, Giulietti et al. revealed key genes involved in pancreatic ductal adenocarcinoma development [6]; Liu et al*.* identified some hub genes in nasal epithelial brushing samples as prognostic biomarkers for allergic asthma [7]; Lin et al*.* revealed IL8 and MMP-9 as key genes for ulcerative colitis [10]; Amrine et al*.* found core biotic stress responsive genes in *Arabidopsis* [8]. However, there is little relevant research focusing on CRC.

In present study, a dataset including 17 human CRC and 17 normal samples in Gene Expression Omnibus (GEO) database was downloaded and the top 5000 genes with big variance were screened out. WGCNA method was adopted to construct a gene co-expression network to find key gene modules closely associated with CRC. The potential function of genes in these key gene modules was analyzed by Gene Ontology (GO) and Kyoto Encyclopedia of Genes and Genomes (KEGG). Meanwhile, the hub genes of each key gene module were identified using Cytoscape software. Then the screened hub genes were further confirmed in UALCAN database. Our results provided the framework of co-expression gene modules of CRC. Meanwhile, the identified key gene modules, key pathways and hub genes in our study may be potential biomarkers and therapeutic targets of CRC, which could promote the clinical diagnosis and treatment of CRC.

## Materials and methods

### Data acquisition and processing

In order to well explore the relationship of gene modules and CRC, gene expression microarray data (GSE32323) including 17 pairs of cancer and non-cancerous tissues from CRC patients and microarray annotation file including information about probes and corresponding genes were obtained from the GEO database (https://www.ncbi.nlm.nih.gov/geo/). Before conducting WGCNA using the gene expression microarray data, we deleted the probes without known gene symbols and probes matching multiple genes. Meanwhile, average expression values of those probes corresponding to one gene were calculated.

### Analysis of microarray data of CRC patients

First, all the cancer and non-cancerous tissues were conducted cluster analysis and the outliers were removed. Then the reserved samples were re-clustered and further correlated with the clinical trait information. In order to conduct the following WGCNA, we selected the top 5000 genes with greater expression co-efficiencies of variation among the samples [9,10].

### Co-expression module construction of CRC

According to the previously reported WGCNA workflow [5], R package of WGCNA was adopted to construct gene co-expression network. To construct the net, we chose a proper soft thresholding power to build the adjacency matrix of the screened 5000 genes. Then the 5000 genes were hierarchically clustered through the topological overlap-based dissimilarity measure. Finally, gene modules were detected by using dynamic tree cut method [11].

### Interaction analysis of co-expression modules and construct module–trait relationship

The eigengenes adjacency based on their correlation was calculated to assess the co-expression similarity of the identified gene modules. Interaction analysis of co-expression modules was performed with *flashClust* [12]. After calculation of the module eigengenes and module significance, we calculated module–clinical trait relationship. Meanwhile, gene modules with high correlation with clinical trait were selected and calculated its module membership and gene significance for clinical trait.

### Functional enrichment analysis of co-expression modules

In order to further investigate the functions and pathways of genes in four key gene modules associated with CRC, GO enrichment analysis and KEGG analysis in DAVID database (https://david.ncifcrf.gov/) were adopted.

### Identification and verification of hub genes

Hub genes are a class of highly connected genes, which have high connectivity with other genes in the same gene module and clinical trait. In order to filter the hub genes in key gene module, we first counted the intra-modular connectivity, and then adopted all the 12 methods in CytoHubba application, a plug-in of Cytoscape. Through calculation and analysis in all 12 methods, the genes of each gene module rank as top ten or twenty in at least eight of the methods were chosen as Hub genes. At last, in order to verify the accuracy of identified hub genes in each gene module, we detected the expression of identified hub genes in normal and cancer group using TCGA-based UALCAN database (http://ualcan.path.uab.edu/analysis.html).

## Results

### Construction of co-expression modules of CRC

**Figure 1:**
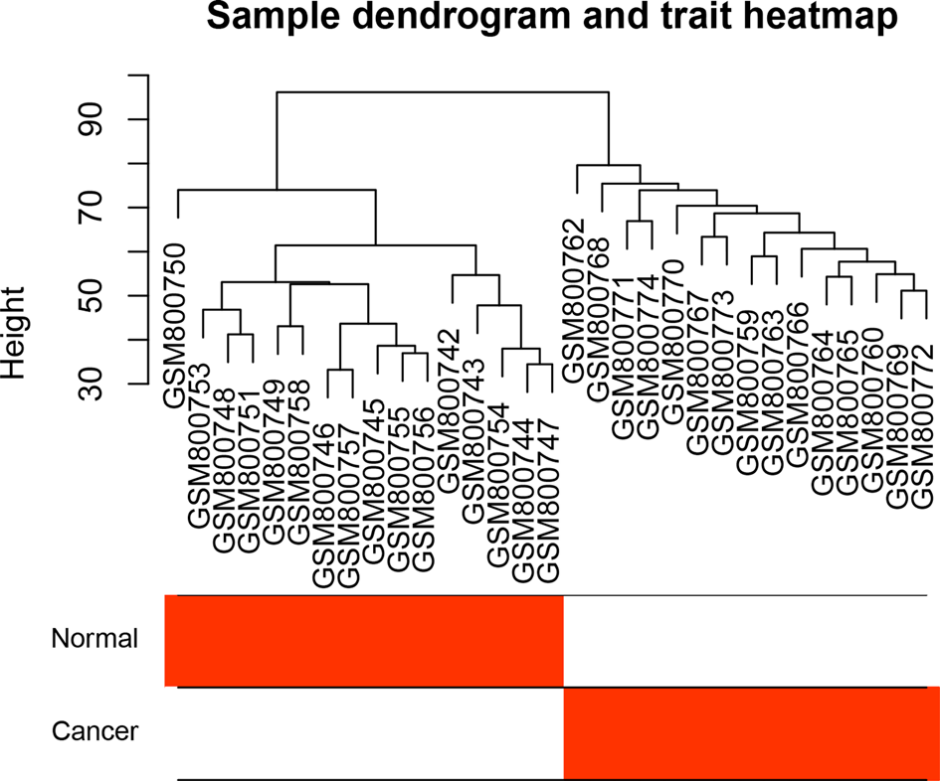
Sample clustering and the trait heatmap to display the sample traits

After processing of the gene expression microarray data, we obtained expression values of 22187 genes totally. Since the gene numbers are always restricted to 3000–5000 during WGCNA, we chose the top 5000 genes with greater variance, which accounted for 22.5% of the total genes obtained. First, we conducted hierarchical clustering analysis on 34 samples by using the expression values of these 5000 genes. The results showed that these samples mainly cluster two groups except for three outlier samples. Thus we reset the parameter ‘Height = 90’ and re-conducted hierarchical clustering analysis. The cluster results showed that the three outliers samples were excluded and the remaining 31 samples were divided into two distinct groups. More obviously, all the cancer tissues clustered into one group and all the normal tissues clustered into the other group (Figure 1), which suggests the relatively large differences on gene expressions between normal tissues and cancer tissues.

**Figure 2:**
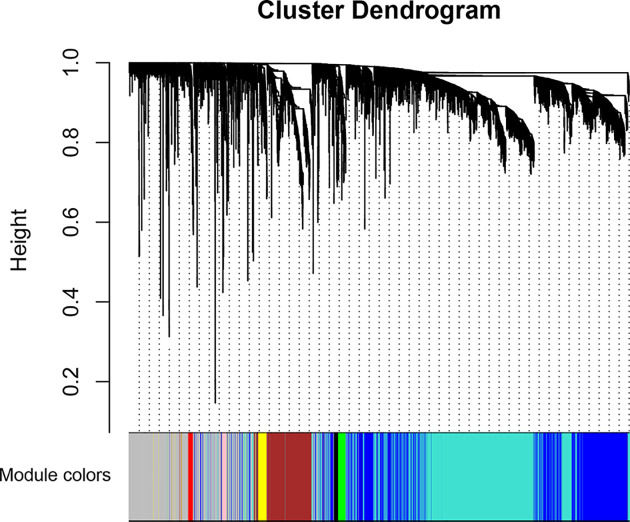
Clustering dendrograms of 5000 genes with dissimilarity based on topological overlap, together with assigned module colors

**Figure 3:**
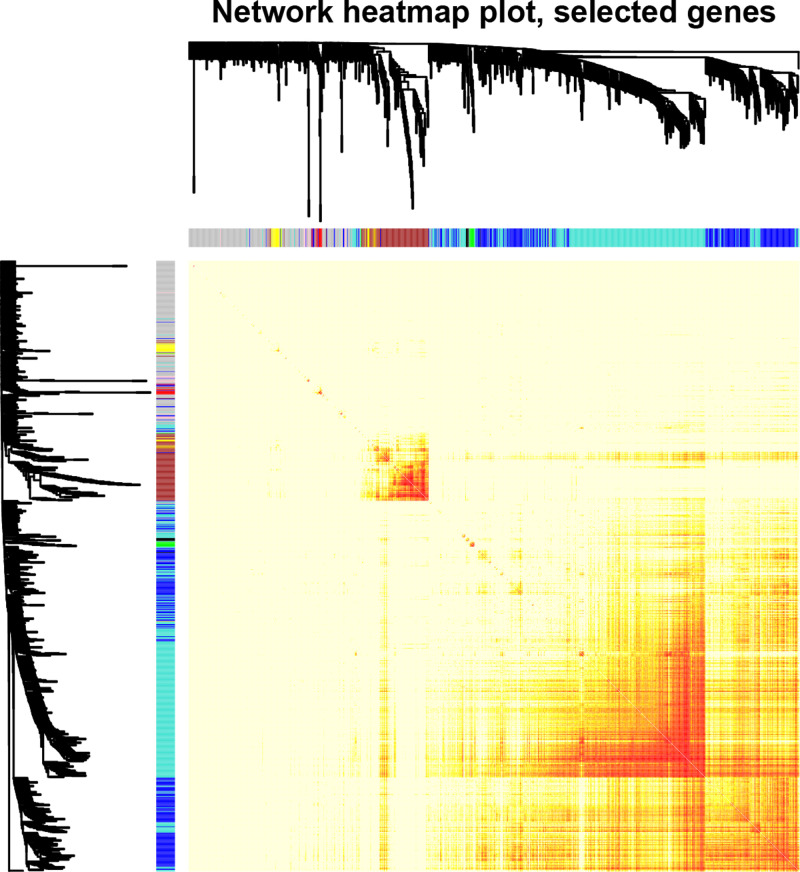
Visualizing 1000 random genes from the network using a heatmap plot to depict the TOM among the genes in the analysis The depth of the red color is positively correlated with the strength of the correlation between the pairs of modules on a linear scale.

A soft thresholding power is important to build an adjacency matrix in WGCNA, so we performed the analysis of network topology for various soft thresholding powers to determine a proper soft thresholding power prior to conducting WGCNA. Our results showed that there was no value less than 15, which could make the scale-free topology fit index reach 0.90 in the unsigned network (data not shown), so we chose an empirical value 14, which was the lowest power that can make the scale-free topology fit index curve flatten out upon reaching a high value (in this case, roughly 0.80), as the soft thresholding power to produce a hierarchical clustering tree of the 5000 genes [13,14]. Ultimately, there were nine gene modules generated, and each one was represented in different color, including black, blue, brown, green, gray, pink, red, turquoise and yellow (Figure 2). These modules and gene numbers they contained were shown in Table 1, among which, the largest turquoise module contained 1780 genes, while the smallest pink module contained 48 genes. In order to detect the interactions between these gene models, we also conducted the network heat map plot of the nine co-expression modules by using 1000 genes, randomly selected (Figure 3). According to the network heatmap plot, each module showed independent validation to each other.

**Table 1:** Identified gene modules and their gene numbers

Gene modules	Gene numbers
Pink	48
Black	49
Red	52
Green	75
Yellow	104
Brown	487
Gray	1072
Blue	1333
Turquoise	1780

### Gene co-expression modules correspond to CRC

**Figure 4:**
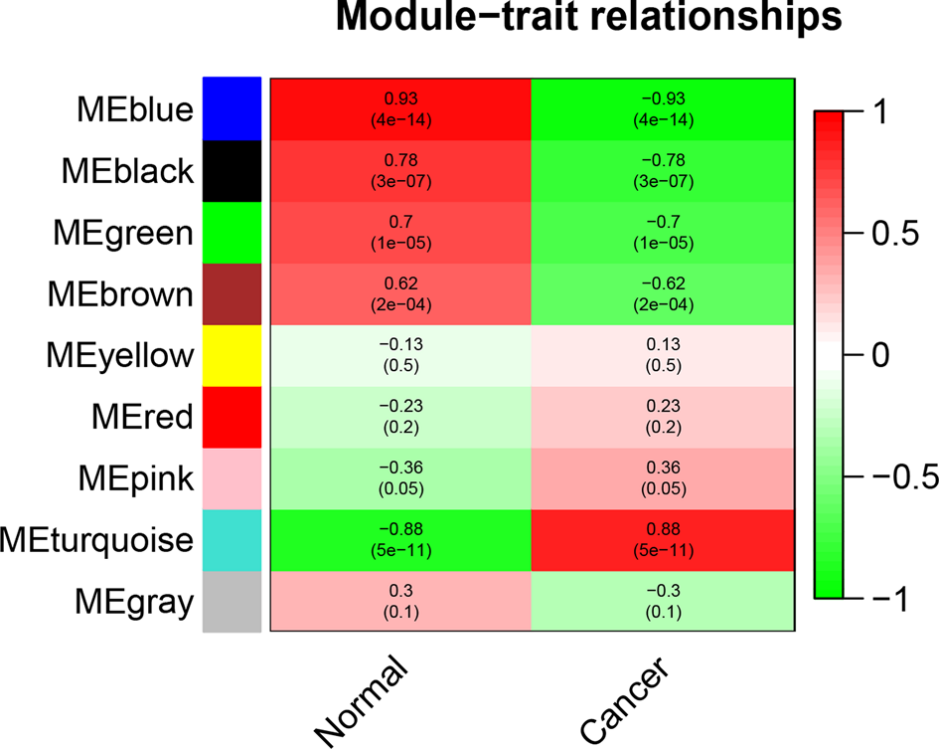
Module–trait relationships Each row corresponds to a module eigengene, each column corresponds to a trait, and each cell consists of the corresponding correlation and *P*-value, which are color-coded by correlated according to the color legend.

**Figure 5:**
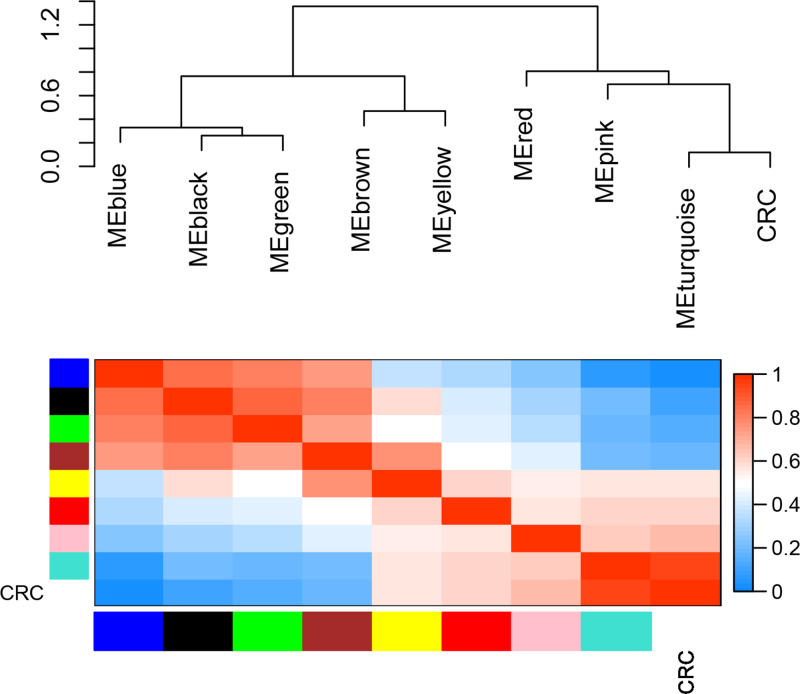
The eigengene dendrogram and heatmap identify groups of correlated eigengenes termed meta-modules The dendrogram indicated that the Turquoise module was highly related to CRC. The heatmap in the panel shows the eigengene adjacency.

**Figure 6:**
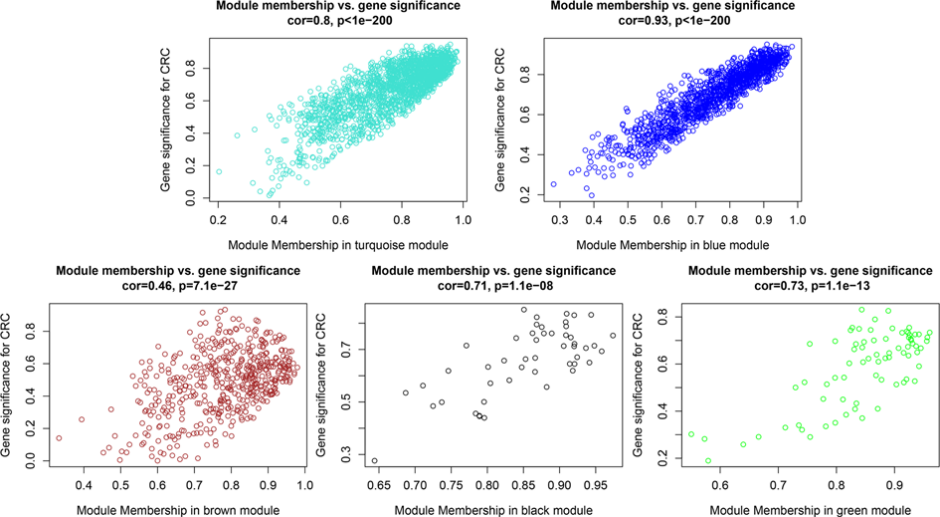
A scatter plot of the gene significance for CRC versus the module membership in the Turquoise, Blue, Brown, Black and Green modules

In the present study, we mainly focused on investigating key gene modules and hub genes associated with CRC. Hence, we investigated the correlations of gene modules and cancer phenotype, which was based on the correlation between module eigengenes and clinical traits. The results showed five of the total nine gene modules were highly associated with CRC, including black (R = 0.78, *P*=3E-07), blue (R = 0.93, *P*=4E-14), brown (R = 0.62, *P*=2E-04), green (R = 0.7, *P*=1E-05) and turquoise (R = −0.88, *P*=5E-11) gene modules (Figure 4). Of which, the turquoise gene module significantly positive correlates with CRC (R = 0.88), while the rest four gene modules (black, blue, brown and green) significantly negatively correlate with CRC. Meanwhile, the eigengene dendrogram and heatmap were also constructed to identify groups of correlated eigengenes associated with CRC, and the results re-verified the correlations of the five identified gene modules with CRC (Figure 5). Eventually, the plots of module membership in different gene modules vs. gene significance further showed that these gene modules have significant correlations with CRC and demonstrate these gene modules are strongly associated with CRC (Figure 6).

### Functional enrichment analysis of genes in interested gene modules

**Figure 7:**
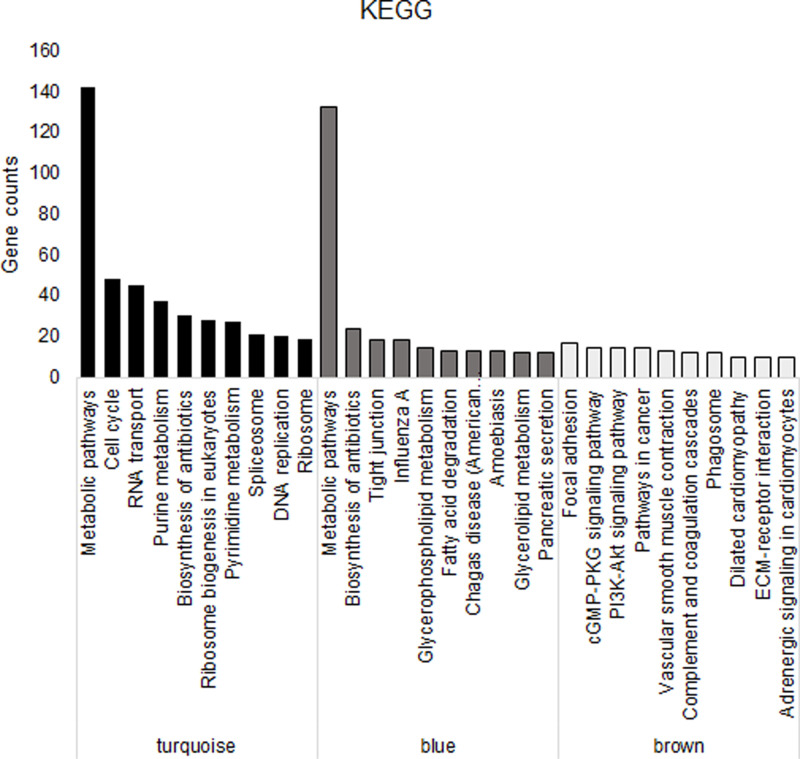
Histograms representing the top ten pathways in identified gene modules by KEGG analysis Black histograms represented the top ten pathways enriched in turquoise gene module. Gray histograms represented the top ten pathways enriched in blue gene module. White histograms represented the top ten pathways enriched in brown gene module.

In order to further understand the main functions and participating pathways of genes included in the identified gene modules significantly correlated with CRC, functional enrichment analysis were conducted, including GO enrichment analysis and KEGG analysis. The top eight to ten biological processes of enrichment in different modules were listed (Table 2) and there was significant difference between different modules. Genes in turquoise module were mainly enriched in biological processes such as cell division, mitotic nuclear division, cell proliferation, DNA replication and repair, protein phosphorylation and cell cycle. Genes in blue module were mainly enriched in biological processes including signal transduction, oxidation-reduction, apoptotic, negative regulation of apoptotic, negative regulation of cell proliferation and inflammatory response. Genes in brown module were mainly enriched in biological processes like cell adhesion, negative regulation of cell proliferation, extracellular matrix organization, muscle contraction and so on. Meanwhile, the result of KEGG analysis (Figure 7) showed that genes included in turquoise module are mainly enriched in metabolic pathways, cell cycle, RNA transport, purine metabolism, biosynthesis of antibiotics, ribosome biogenesis in eukaryotes, pyrimidine metabolism and so on. Similarly, genes in blue module also enriched in metabolic pathways and biosynthesis of antibiotics. Besides, they were also enriched in other pathways, such as tight junction, fatty acid degradation, glycerophospholipid and glycerolipid metabolism. Genes included in blue module relatively balanced enrichment in various pathways, including focal adhesion, cGMP-PKG signaling, PI3K-Akt signaling and pathways in cancer.

**Table 2:** The top biological processes of enrichment in different modules

Gene module	Biological processes	Counts	*P*-value
Turquoise	Cell division	114	3.21E-37
	Mitotic nuclear division	80	7.00E-26
	DNA replication	56	6.95E-21
	Sister chromatid cohesion	42	5.25E-18
	G_1_/S transition of mitotic cell cycle	38	8.51E-15
	rRNA processing	51	4.62E-11
	DNA replication initiation	18	1.16E-10
	G_2_/M transition of mitotic cell cycle	36	3.07E-09
	DNA repair	49	1.29E-08
	Cell proliferation	66	1.39E-08
Blue	O-glycan processing	16	3.37E-06
	Establishment of protein localization to plasma membrane	12	4.27E-05
	Epithelial cell differentiation	15	1.04E-04
	Neural tube closure	15	2.98E-04
	Excretion	10	3.88E-04
	Positive regulation of establishment of protein localization to plasma membrane	9	4.17E-04
	Odontogenesis of dentin-containing tooth	12	5.61E-04
	Negative regulation of cell proliferation	43	7.11E-04
	Post-embryonic camera-type eye development	4	9.61E-04
	Negative regulation of apoptotic process	47	0.001125
Brown	Muscle contraction	19	5.43E-11
	Extracellular matrix organization	23	1.32E-09
	Cell adhesion	31	4.54E-07
	Positive regulation of cell–substrate adhesion	9	2.26E-06
	Negative regulation of cell proliferation	25	2.30E-05
	Platelet degranulation	12	2.98E-05
	Mesenchyme migration	4	1.24E-04
	Cell junction assembly	5	1.27E-04
	Neuron migration	11	1.79E-04
	Complement activation, alternative pathway	5	1.80E-04

### Identified and verified hub genes of gene modules associated with CRC

A hub gene, which often refers to a gene that has many interactions with other genes in gene networks, usually plays an essential role in gene regulation and biological processes [15,16] Intra-modular connectivity was calculated for each gene and those with high intra-modular connectivity were considered hub genes [5]. Eight types of algorithms in CytoHubba, a plug-in of Cytoscape software, were adopted to better identity hub genes in the modules associated with CRC. For turquoise, black, blue and brown gene modules, only those genes ranked the top 25 in at least seven types of algorithms were identified as hub genes. While for green gene module, those genes ranked in the top 15 in at least seven algorithms were identified as hub genes.

**Figure 8:**
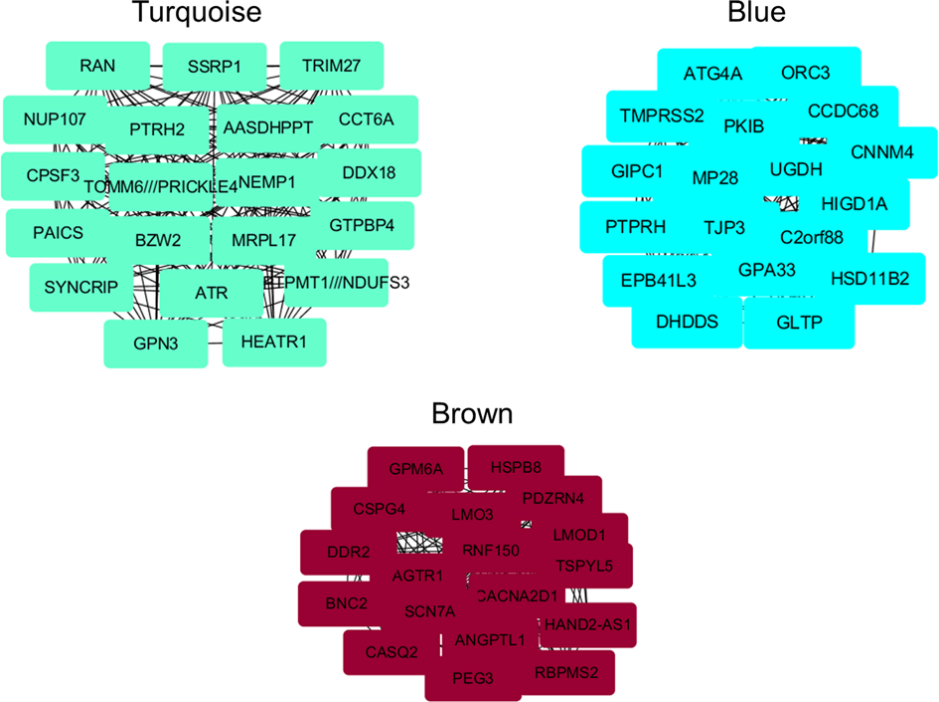
The identified hub genes in identified gene modules using Cytoscape software Turquoise represented the identified hub genes enriched in turquoise gene module. Blue represented the identified hub genes enriched in blue gene module. Brown represented the identified hub genes enriched in brown gene module.

In the turquoise gene module, 20 hub genes were identified positively correlated with CRC, including *PAICS, ATR, AASDHPPT, DDX18, NUP107, TOMM6, GTPBP4, RAN, GPN3, SYNCRIP, CPSF3, PTRH2, SSRP1, BZW2, NEMP1, PTPMT1, CCT6A, HEATR1, TRIM27* and *MRPL17* (Figure 8). Meanwhile, lots of hub genes were identified negatively correlated with CRC in the blue and brown gene modules (Figure 8). Nineteen hub genes in the blue gene module were identified negatively correlated with CRC, including *DHDDS, ORC3, HIGD1A, GIPC1, CNNM4, UGDH, GLTP, EPB41L3, PTPRH, HSD11B2, ATG4A, C2orf88, CCDC68, TMPRSS2, TJP3, PKIB, MMP28* and *GPA33*. In the black gene module, *IGLC1, MZB1, IGKV1OR2-108, DNASE1L3, IGLL5* and *CPA3* were identified negatively correlated with CRC. Ten hub genes, including *MAP4K1, PAX5, PVRIG, P2RY8, SELL, TBC1D10C, VPREB3, NAPSB, CCR7* and *TCL1A*, in the green gene module were identified negatively correlated with CRC. Eighteen hub genes in the brown gene module were identified negatively correlated with CRC, including *HAND2-AS1, LMO3, AGTR1, PDZRN4, RBPMS2, LMOD1, GPM6A, CASQ2, CACNA2D1, RNF150, ANGPTL1, HSPB8, SCN7A, CSPG4, DDR2, BNC2, TSPYL5* and *PEG3*.

**Figure 9:**
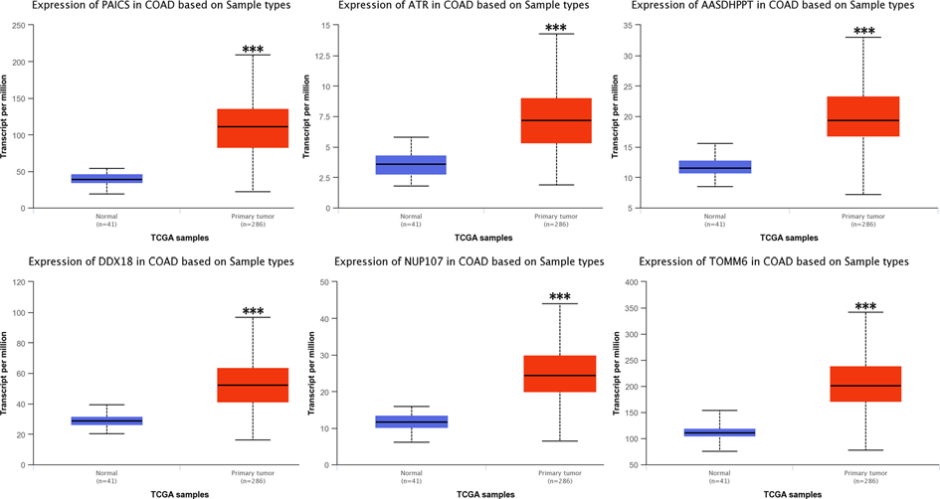
The expressions of several hub genes identified from turquoise gene module in colon cancer and normal samples of UALCAN database The expressions of PAICS, ATR, AASDHPPT, DDX18, NUP107 and TOMM6 in colon cancer (red color) and normal samples (blue color) of UALCAN database. ***P<0.001.

Furthermore, the UALCAN database (http://ualcan.path.uab.edu/analysis.html), which is an analyzing platform based on TCGA database, was used to verify the expression of sorted hub genes in various modules associated with CRC in colon adenocarcinoma. In turquoise gene module, the only gene module positively correlated with CRC, the top ranked hub genes were all verified to be expressed significantly highly in colon cancer compared with normal, including *PAICS* (*P*=1.62E-12), *ATR* (*P*<1E-12), *AASDHPPT* (*P*=1.62E-12), *DDX18* (*P*<1E-12), *NUP107* (*P*<1E-12) and *TOMM6* (*P*<1E-12) (Figure 9). Similarly, the top ranked hub genes in other gene modules negatively correlated with CRC were also verified to be expressed significantly lower in colon cancer when compared with normal, such as *DHDDS* (*P*<1E-12), *HIGD1A* (*P*=1.67E-15), *GIPC1* (*P*=4.98E-11), *CNNM4* (*P*=8.66E-13) in the blue gene module, *DNASE1L3* (*P*=1.63E-12) and *CPA3* (*P*<1E-12) in the black gene module (data not shown).

## Discussion

WGCNA is an analytical method mainly focusing on the association between co-expression gene modules and clinical traits. Due to its distinct advantages, WGCNA has been widely utilized to discover the synergetically expressed modules and candidate key hub genes associated with various diseases, such as allergic asthma [7], T-cell lymphoma [17], osteosarcoma [18], hepatocellular carcinoma [19], uveal melanoma [20] and other cancers. While, there is almost no relevant paper adopting WGCNA approach to investigate hub genes associated with CRC pathogenesis.

CRC has become one of the most common worldwide cancers and a major cause of cancer deaths. Due to its heterogeneity and complexity, there are few effective therapeutic methods for CRC [21]. Therefore, it is urgent to discover new key genes as therapeutic targets to provide new methods and ideas for treatment of CRC. The main objective of the present study is to identify key gene modules and detect key hub genes that could be regarded as therapeutic targets of CRC. In order to achieve this goal, we utilized a global and advanced analytical approach—WGCNA to find key gene modules associated with the pathogenesis of CRC by constructing a gene co-expression network. Furthermore, Cytoscape was adopted to screen out candidate key genes of gene modules associated with molecular pathogenesis of CRC.

In the present paper, the hierarchical clustering analysis result showed that 31 samples were divided into two distinct groups, of which, all the normal samples were classed into the normal group and all the cancer samples were classed into the CRC group, which suggests that there are obvious differences between the gene expression profiles of normal and CRC samples. Next, we constructed gene co-expression networks and totally found nine gene modules by analyzing 5000 genes of 31 samples with WGCNA approach. Meanwhile, the network heat map plot showed that all the identified gene modules had almost no correlation with each other. Through analyzing the correlations between identified gene modules and CRC, we found that there was a strong positive correlation between the turquoise module and CRC and that four gene modules (black, blue, brown and green) were negatively correlated with CRC. As there are few genes in both black and green modules, we mainly focused on the other three gene modules to investigate the functions of these gene modules. KEGG pathway analysis showed that there were similarities and differences on their functions of the three gene modules. As the only gene module significantly positively correlated with CRC, genes in turquoise gene module mainly involved in metabolic pathway, cell cycle, RNA transport, purine metabolism, biosynthesis of antibiotics etc. Similarly, genes in the blue gene module were also mainly enriched in metabolic pathway, which suggests that changes in gene expressions in metabolic pathway may play vital roles in the pathogenesis of CRC.

For a better understanding of the relationship between gene modules and CRC, Cytoscape was used to screen out the hub genes in each CRC-related gene modules. Lots of hub genes were identified in turquoise, blue and brown gene modules. To further test and verify whether the expressions of these identified hub genes were correlated with CRC and could be regarded as candidate biomarkers or therapeutic targets of CRC, we investigated the expressions of these identified hub genes in normal and CRC tissues in UALCAN database, which based on TCGA database. Results confirmed that these hub genes in turquoise module, including *PAICS, ATR, AASDHPPT, DDX18, NUP107* and *TOMM6* were all expressed significantly higher in CRC group compared with normal group. These results were somewhat consistent with those of previous researches. Bianchini et al. found PAICS was one of the most significant genes which was up-regulated in CRC patients [22]. Meanwhile, PAICS has also been suggested playing vital roles in pathogenesis of other cancers, including bladder cancer and lung cancer [23,24]. ATR protein kinase has been proved as a key enzyme in the DNA damage response (DDR) which can limit the efficacy of cytotoxic chemotherapy agents and radiation during cancer treatment [25]. Therefore, it has been used as a cancer therapeutic target for its specific inhibitors [26]. AASDHPPT showed the strongest signal-to-noise ratio in ascites fluid pooled from ovarian cancer patients by using protein microarrays [27]. All these previous results laterally demonstrated the accuracy of the hub genes we identified, which suggested these hub genes may play key roles in CRC and could be potential biomarkers and therapeutic targets of CRC. Besides, the top ranked hub genes in gene modules negatively correlated with CRC were also verified to be expressed significantly lower in CRC. Combining the confirmed hub genes in turquoise and other gene modules would be a better method for discrimination between CRC and healthy subjects.

In the present study, five gene modules were identified significantly correlated with the pathogenesis of CRC by WGCNA analysis. The turquoise co-expression gene module was significantly positively, and other four gene modules were negatively correlated with CRC. Meanwhile, by analyzing the pathways and functions, we found genes in main gene modules were mainly involved metabolic pathways. Further, we also identified key hub genes positively correlated with CRC in the turquoise gene module and verified some of these hub genes in UALCAN database. These hub genes identified may be potential biomarkers for diagnosis and treatment of CRC and need further studies to clarify their roles in CRC.

## Abbreviations

CRC: colorectal cancer
cGMP-PKG: cyclic guanosine 3′, 5′-monophosphate-protein kinase G
DAVID: Database for Annotation, Visualization and Integrated Discovery
GEO: gene expression omnibus
GO: gene ontology
KEGG: Kyoto Encyclopedia of Genes and Genomes
TCGA: The Cancer Genome Atlas
WGCNA: weighted gene co-expression network analysis
